# Programming Failure Mode Transitions in Polyurea-Reinforced 3D-Printed ABS and PA-GF Cellular Metamaterial Composites

**DOI:** 10.3390/polym18121466

**Published:** 2026-06-11

**Authors:** Rodrigo Valle, César Garrido, Víctor Tuninetti

**Affiliations:** 1 Construction Multidisciplinary Research Group, Facultad de Arquitectura, Construcción y Medio Ambiente, Universidad Autónoma de Chile, Talca 3460000, Chile; rodrigo.valle@uautonoma.cl; 2 Department of Mechanical Engineering, Universidad del Bío-Bío, Concepción 4081112, Chile; 3 Department of Mechanical Engineering, Universidad de La Frontera, Temuco 4811230, Chile

**Keywords:** Design for Additive Manufacturing (DfAM), hybrid prototyping, architected cellular materials, interpenetrating phase composites (IPC), failure mode transition

## Abstract

Additively manufactured cellular architectures frequently exhibit brittle failure under impact due to layer-induced stress concentrations. Through the programming of architectural and material design, specifically combining Fused Deposition Modeling (FDM) lattice topology with hyperelastic polyurea infiltration, this study achieves active control over the macroscopic transition from catastrophic structural fragmentation to stable progressive collapse. To evaluate this, auxetic and honeycomb specimens printed with ABS and glass-fiber-reinforced polyamide (PA-GF) were evaluated in unreinforced and polyurea-infiltrated states under quasi-static compression, three-point bending, and Charpy impact loading. Results show that the compressive response depends primarily on cellular topology; the pure auxetic (A-A) configuration provided the highest stiffness and energy absorption. Polyurea infiltration did not significantly alter elastic stiffness but increased post-yield stability, leading to a 96.6% elastic recovery in PA-GF A-A structures. In flexure, the base polymer governed stiffness, with ABS structures measuring 68% stiffer than PA-GF. Unreinforced ABS achieved 34% higher specific energy absorption (SEA) than PA-GF under compression, with the A-H topology maximizing SEA. Under dynamic impact, PA-GF absorbed an average of 70% more energy than ABS, and the H-A configuration recorded the highest impact resistance. The addition of polyurea shifted the failure mode from brittle fragmentation to stable elastomeric deformation, increasing absorbed impact energy by 52% for ABS and over 30% for PA-GF, preventing catastrophic structural failure. Integrating topological sequencing with elastomeric confinement provides a direct method to control energy dissipation and damage tolerance in 3D-printed cellular composites.

## 1. Introduction

Over the last decade, additive manufacturing (*AM*) technologies have undergone remarkable development, enabling the manufacture of complex geometries that were previously unfeasible with conventional manufacturing methods. In particular, fused deposition modeling (*FDM*) has become one of the most widely adopted *AM* techniques due to its accessibility, material versatility, and its ability to enable rapid prototyping and functional part production [1]. Continuous improvements in print accuracy, filament formulations, and process control have significantly expanded the scope of *FDM* applications, enabling its use in the manufacture of structural components [2], energy absorption systems [3], and sandwich panels [4].

In this context, cellular structures manufactured using additive manufacturing have attracted increasing attention as a design strategy for developing new lightweight structures with customized mechanical properties [5,6]. By controlling parameters such as cellular topology, relative density, and material distribution, cellular architectures allow for the manipulation of stiffness [7], strength [8], and energy absorption [9]. The geometric freedom offered by 3D printing allows the fabrication of periodic lattices for the development of new architectural materials with optimized load and deformation characteristics, positioning these structures as promising candidates for applications in impact mitigation [10,11], vibration control [12], and structural protection [13]. Among the wide range of cellular architectures, auxetic structures and conventional honeycomb lattices have been extensively researched due to their distinctive mechanical responses. Auxetic structures, characterized by a negative Poisson’s ratio, exhibit lateral contraction under compression, which promotes greater indentation resistance [14], shear stiffness [15], and energy absorption [16]. In contrast, honeycomb structures offer high stiffness-to-weight ratios and efficient load transfer mechanisms [17,18,19]. Recent studies have explored both pure auxetic and honeycomb configurations, as well as hybrid combinations of these topologies, demonstrating that the interaction between auxetic and honeycomb mechanisms can be exploited to tune stiffness, collapse modes, and energy dissipation capacity under quasi-static and dynamic loading conditions [20,21].

Despite the potential of these cellular structures, manufacturing them using *FDM* imposes inherent limitations that limit their energy absorption capacity [22]. The layer-by-layer deposition process produces anisotropic mechanical behavior and weak interlaminar bonding, often resulting in brittle fracture and premature failure under compressive, flexural, or impact loads [23]. This limitation is particularly critical for energy absorption applications, where stable, damage-resistant plastic deformation is required [24]. Therefore, the literature shows various strategies to overcome these disadvantages, including cementitious reinforcements [25], to reinforcements of cellular architectures with secondary phases such as thermosetting resins [26], polymeric foams [27] and polyurethane elastomers [28,29]. For instance, Jiang et al. [30] improved the damping performance of auxetic honeycomb sandwich plates by combining the structure with higher-density foam cores that increase natural frequencies and damping loss factors. Similarly, ref. [31] proposed a reentrant auxetic honeycomb reinforced with recycled sugarcane bagasse fibers and rubber particles, demonstrating that fiber-rubber hybridization significantly improves toughness. Other studies [32] demonstrate that in both conventional and auxetic honeycomb sandwich beams, polyurethane foam filling significantly improves explosion resistance and specific energy absorption through a balanced enhancement in flexural strength. In [33,34], filling a laser-selectively melted auxetic honeycomb with polyurethane foam was shown to significantly improve in-plane compressive strength and energy absorption, driven by the interaction between auxetic kinematics and the foam matrix. Additionally, ref. [35] demonstrated that thin-walled aluminum tubes filled with a laterally reinforced honeycomb core achieve substantial improvements in crush performance, while [36] identified recessed honeycomb architectures as the most effective auxetic topology for impact applications when combined with ultra-high-performance concrete. Finally, while advanced metallic metamaterials fabricated via laser powder bed fusion have demonstrated ultrahigh damping efficiencies [37], their complex manufacturing requirements highlight the ongoing need for accessible, damage-tolerant polymeric alternatives.

Despite the growing number of studies on reinforced cellular structures [38,39,40,41], the fundamental mechanical distinction between conventional rigid fillers, standard polyurethane foams, and hyperelastic polyurea remains largely unarticulated in the context of FDM composites. While rigid matrices (e.g., epoxy, cementitious pastes) significantly enhance absolute compressive strength, they typically exacerbate catastrophic brittle fracture under dynamic impact. Conversely, standard polyurethane foams improve damping and energy absorption but often fail to provide sufficient interfacial confinement to prevent the internal 3D-printed skeleton from fracturing prematurely [30,32]. Consequently, the use of polyurea as a hyperelastic elastomeric reinforcement for additively manufactured auxetic and honeycomb structures remains largely unexplored. Furthermore, there is a lack of systematic studies addressing how the mechanical response of these architectural materials evolves under different loading regimes, particularly when comparing quasi-static and dynamic conditions. The combined effect of integrating two mechanically different constituents, a relatively rigid polymeric structure and a flexible elastomeric matrix as reinforcement, has not been thoroughly investigated. This is especially true regarding how this material synergy influences stiffness, strength, energy absorption, damage tolerance, and elastic recoverability under loading conditions. Therefore, this paper presents a comprehensive experimental study to evaluate the mechanical behavior of auxetic, honeycomb, and auxetic–honeycomb hybrid cellular structures made from *ABS-FRO* and *PA-GF* filaments, with and without polyurea reinforcement. The primary objective of this research is not to optimize for lightweight Specific Energy Absorption (SEA), but to overcome the intrinsic layer-induced brittleness of FDM through a hybrid prototyping methodology. The novelty lies in combining perimeter-based FDM printing with cold-cast polyurea infiltration to decouple stiffness from toughness, shifting the paradigm from simply increasing structural strength to actively programming a failure mode transition—from catastrophic structural fragmentation to stable elastomeric survival.By filling this research gap, the present study provides new insights into the Design for Additive Manufacturing (DfAM) of damage-tolerant, highly recoverable energy-absorbing structures.

The remainder of this article is organized as follows. Section 2 describes the design of the cellular structures, the materials used, and the experimental procedures adopted for quasi-static compression, three-point bending, and Charpy impact tests. Section 3 presents and discusses the experimental results, analyzing the influence of cellular topology, material selection, and polyurea reinforcement on the mechanical response under different loading conditions. Finally, Section 4 summarizes the main findings and conclusions of the study, highlighting the implications of the results for the design of lightweight, energy-absorbing cellular structures.

## 2. Materials and Methods

### 2.1. Design of Cellular Architectures

**Figure 1:**
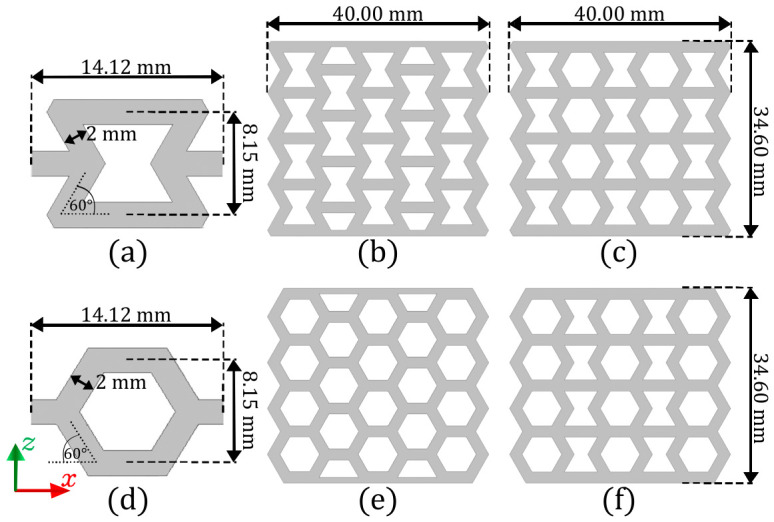
Cross-section of the experimental specimens for quasi-static compression. The configurations include: (**b**) pure Auxetic (A−A), (**e**) pure Honeycomb (H−H), (**c**) Auxetic–Honeycomb hybrid (A−H), and (**f**) Honeycomb–Auxetic hybrid (H−A). Subfigures (**a**) and (**d**) show the unit cells of the Auxetic and Honeycomb structures, respectively.

The cellular structures evaluated in this research comprise four distinct configurations: pure Re-entrant Auxetic (A−A), pure Honeycomb (H−H), and two hybrid configurations that combine auxetic and honeycomb unit cells in sequence: Auxetic–honeycomb (A−H) and Honeycomb–Auxetic (H−A). This design approach is similar to that proposed in [42]; however, the present work extends the analysis by comparing energy absorption capacity across various experimental conditions using four different 3D printing materials. The Auxetic Structure was based on a unit cell with a re-entrant angle of $\theta =60^{{}^{\circ}}$, known for its negative Poisson’s ratio and high stiffness [43,44], which also promotes high levels of energy absorption under compressive [45] and impact loading [46,47]. In contrast, the honeycomb geometry followed a conventional hexagonal pattern, with an outgoing angle of $\theta =60^{{}^{\circ}}$. Hybrid configurations were designed to investigate the potential synergistic effects arising from combining these geometries: the A−H configuration integrates auxetic cells on the impact side, followed by honeycomb cells. In contrast, the H−A configuration reverses this order. All geometries were parameterized to ensure identical external dimensions, consistent relative densities, and comparable unit cell sizes. Similarly, the wall thicknesses of all structures are the same t=2 mm, which allowed a fair comparison between the configurations. The specific unit cells for the auxetic and honeycomb architectures are detailed in Figure 1a and Figure 1d, respectively. Figure 1b shows the pure Auxetic (A−A) configuration, while Figure 1e represents the pure Honeycomb (H−H). The hybrid configurations include the Auxetic–Honeycomb (Figure 1c) and the Honeycomb–Auxetic (Figure 1f). To fabricate these configurations, extrusion was performed up to a height of 40 mm to achieve the three-dimensional structure.

**Figure 2:**
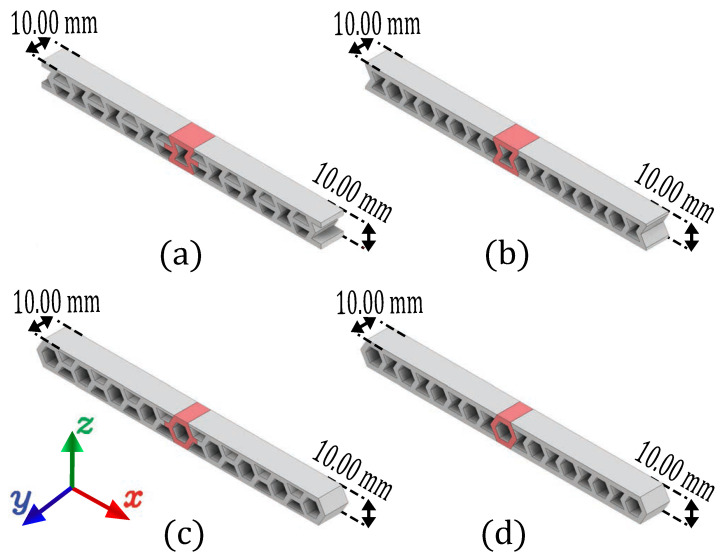
Experimental specimens for bending and impact tests: (**a**) pure Auxetic (A−A), (**b**) Auxetic–Honeycomb hybrid (A−H), (**c**) pure Honeycomb (H−H) and (**d**) Honeycomb–Auxetic hybrid (H−A).

Similarly, Figure 2 shows the experimental beams designed based on the same geometric configurations presented in Figure 1, corresponding to the pure Auxetic (A−A, Figure 2a), hybrid Auxetic–Honeycomb (A−H, Figure 2b), pure Honeycomb (H−H, Figure 2c), and hybrid Honeycomb–Auxetic (H−A, Figure 2d) structures. In this case, the geometries were adapted to form prismatic specimens suitable for three-point bending and Charpy pendulum impact tests. All beam specimens were designed with a constant length of 124.64 mm, ensuring consistent boundary conditions and allowing direct comparison of structural response. These configurations enable systematic evaluation of mechanical performance under bending and impact loading, assessing the influence of cellular topology on stiffness, energy absorption capacity, flexural stability, and impact fracture resistance.

For each configuration, three sets of specimens were produced for compression, bending, and impact tests, with each set produced in identical pairs. One group remained unfilled to assess the intrinsic mechanical response, while the other was reinforced with cold polyurea. This pairing enables a direct comparison of the elastomeric reinforcement’s effect on energy absorption, stiffness, and impact resistance, strengthening the validity of the experimental conclusions.

### 2.2. Experimental Procedure

**Figure 3:**
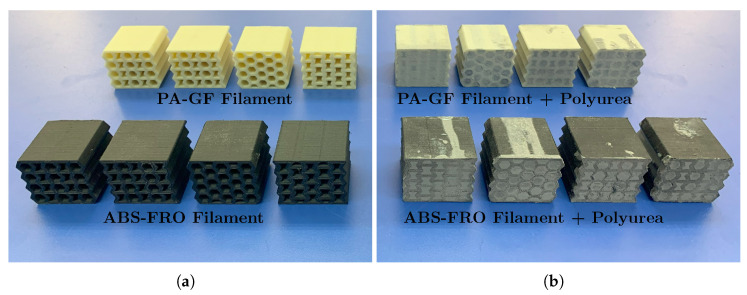
Manufacture of experimental specimens for compression testing using FDM printing with *PA-GF* and *ABS-FRO* filaments (**a**) and structures reinforced with cold polyurea (**b**).

**Figure 4:**
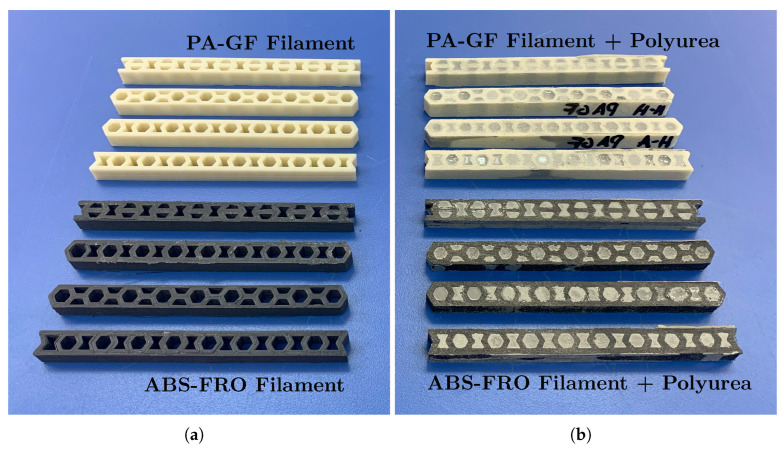
Manufacturing of experimental samples for flexion and Charpy pendulum impact testing using FDM printing with *PA-GF* and *ABS-FRO* filaments (**a**) and structures reinforced with cold polyurea (**b**).

All hybrid prototypes were manufactured on the xz plane using a *Creality K1C* FDM printer (Shenzhen Creality 3D Technology Co., Ltd., Shenzhen, China). The geometric configurations shown in Figure 1 were printed using *Silver 3D* glass-fiber-reinforced polyamide (*PA-GF*) and Acrylonitrile Butadiene Styrene *ABS-FRO* filaments. The resulting printed structures for compression are shown in Figure 3a, while the beams for flexural and impact testing are shown in their unreinforced state in Figure 4a.

The fused deposition process was carried out at an extrusion temperature of 250 °C for *PA-GF* and 300 °C for *ABS-FRO*, maintaining a bed temperature of 100 °C in both cases. To ensure structural consistency and minimize void formation, all prototypes were printed using a 0.4 mm nozzle, a layer height of 0.2 mm, and a constant printing speed of 50 mm/s. The struts were printed with 100% infill utilizing concentric perimeter lines to align the polymer extrusion paths with the longitudinal axis of the cell walls. This xz-plane orientation was strategically selected to mitigate the inherent layer-by-layer anisotropy of FDM across all evaluated loading configurations. By aligning the raster lines with the primary load-bearing struts, the applied forces during quasi-static compression, three-point bending, and dynamic impact are distributed predominantly parallel to the extrusion paths. This arrangement minimizes out-of-plane tensile stresses across the inter-raster interfaces, thereby suppressing premature interlayer delamination, the most common initiator of brittle fracture in 3D-printed polymers. Consequently, this specific build orientation ensures that the structural failure sequence and energy dissipation mechanisms are governed genuinely by the kinematics of the cellular topology and the hyperelastic polyurea confinement, rather than being prematurely truncated by FDM manufacturing defects.

To evaluate the influence of elastomeric reinforcement on the mechanical response of the cellular structures, the interpenetrating phase composite was produced using a two-component polyurea system, manually mixed with its corresponding catalyst at a volumetric ratio of 4.8:1 until a visually homogeneous mixture was achieved.

Before infiltration, the polyurea was mechanically characterized under uniaxial tensile loading using a Zwick/Roell Z100 universal testing machine. For this purpose, the mixed polyurea system was allowed to cure for 24 h under ambient laboratory conditions to ensure complete polymerization before specimen testing. Ten specimens were prepared according to the dimensional recommendations of ASTM D638 and tested at a crosshead displacement rate of 50 mm/min. The tensile response exhibited a yield strength of 4.5±0.2 MPa, an ultimate tensile strength of 15.5±1.2 MPa, maximum elongation of approximately 500%, and a shore A hardness of 66.4±1.3, confirming the highly deformable and energy-dissipative nature of the elastomeric phase.

Subsequently, the liquid polyurea was introduced into the cellular voids of the 3D-printed prototypes by manual cold-cast infiltration. This low-pressure infiltration procedure enabled complete filling of the internal cavities while avoiding thermally induced distortion of the thermoplastic skeleton. During the filling process, the samples were gently tapped to minimize air entrapment and promote a uniform distribution of the elastomeric phase. Finally, excess polyurea was removed from the external surfaces before curing.

The infiltration process for the compression samples is illustrated in Figure 3b, and the final polyurea-reinforced beams are shown in Figure 4b. All reinforced samples were cured at room temperature for 24 h to ensure complete polymerization and stable mechanical properties before testing. This reinforcement strategy resulted in a hybrid structure in which the rigid network of the cellular structure interacts with the highly deformable elastomeric phase, enabling improved energy dissipation under mechanical loading.

### 2.3. Experimental Repeatability and Manufacturing Robustness

To demonstrate the reliability of the fabrication process, controlled experiments using the A-H configuration with ABS-FRO were conducted. Five independent replicates were fabricated and tested under quasi-static compression to assess experimental repeatability.

**Figure 5:**
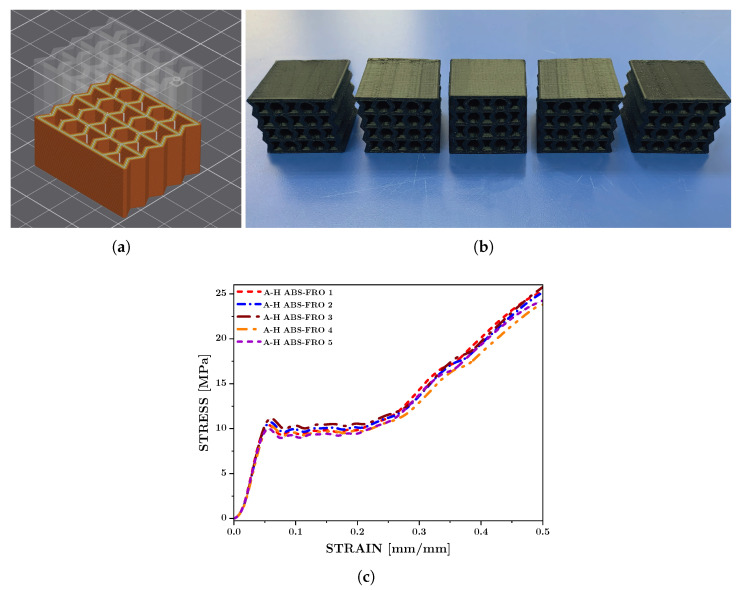
Experimental analysis of the repeatability of the A-H lattice configuration manufactured with ABS-FRO. (**a**) Perimeter-based printing strategy. (**b**) Five replicas were used for compression tests. (**c**) Stress–strain curves from the compression tests.

Figure 5a illustrates the perimeter-based printing strategy adopted in this study. Unlike conventional FDM approaches that rely on internal rasterized infill patterns, all structures were fabricated using continuous perimeter extrusion paths aligned with the geometry of the cellular struts. In this configuration, the deposited filaments follow longitudinal paths along the load-bearing elements, producing continuous structural paths rather than discontinuous infill interfaces. This strategy minimizes common sources of variability in the fabrication of lattice structures via FDM, including the effects of raster orientation, infill heterogeneity, and weak interlayer discontinuities. Consequently, mechanical loads are transmitted predominantly along continuous filament paths, significantly reducing anisotropic effects and improving structural consistency.

Figure 5b shows the five independent specimens fabricated for the repeatability analysis. At the same time, the corresponding stress–strain curves obtained from the compression tests are presented in Figure 5c. A high degree of agreement is observed among all the experimental curves, indicating excellent repeatability of the deformation response and highly stable collapse mechanisms. To quantitatively evaluate the experimental consistency, the main mechanical properties derived from the compression tests were statistically analyzed. Table 1 summarizes the mean value, standard deviation (SD), and coefficient of variation (CV) obtained for Young’s modulus, the elastic limit, and specific energy absorption (SEA).

The results reveal extremely low experimental dispersion, with CV values ranging from 2.09% to 4.31%. In particular, Young’s modulus and the elastic modulus showed variations of less than 3%. In contrast, the elastic limit remained below 5%, confirming the excellent manufacturing consistency achieved through the adopted perimeter-based manufacturing strategy. These values are substantially lower than the variability typically observed in cellular structures manufactured using FDM, where the mechanical response is frequently affected by the stochastic distribution of infill and interlayer defects.

Based on the demonstrated repeatability and the highly consistent mechanical response observed across replicated specimens, a single representative specimen was fabricated and tested for each evaluated topology, material system, and reinforcement condition. This approach enabled a direct comparison of configurations, minimizing unnecessary material consumption and experimental redundancy while introducing minimal uncertainty into the mechanical analysis.

### 2.4. Mechanical Testing Protocols

**Figure 6:**
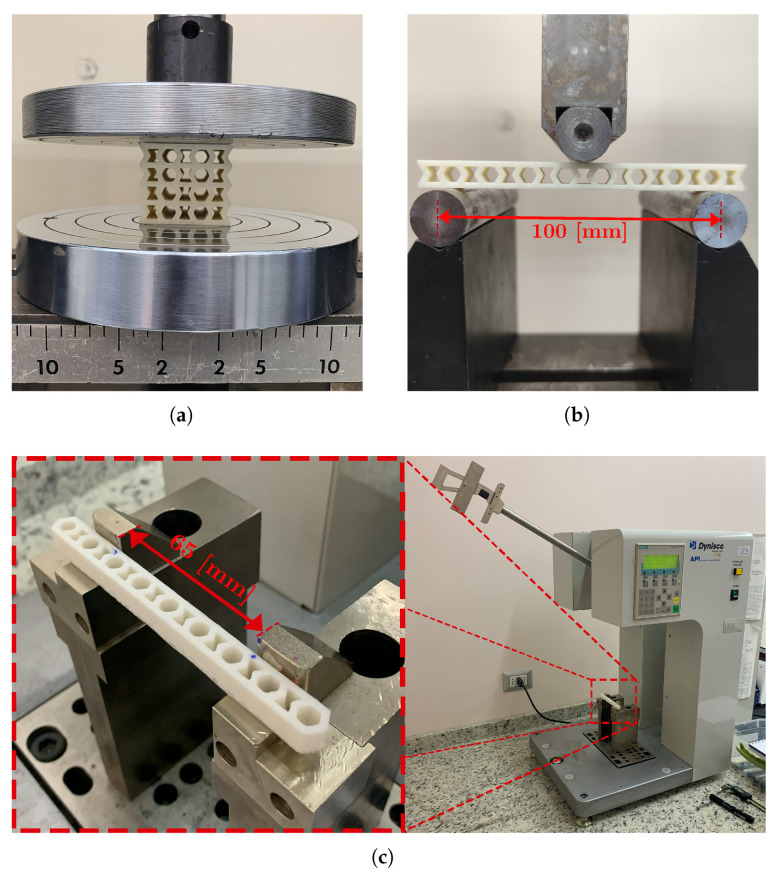
Experimental setup for the mechanical tests performed in this study. (**a**) Quasi-static compression tests were conducted on all cellular structures to evaluate their load–displacement and energy-absorption behavior. (**b**) Three-point bending tests are used to assess flexural stiffness and failure mechanisms. (**c**) Charpy pendulum impact tests were carried out to determine the impact energy and fracture response of the specimens.

All mechanical tests were performed under controlled laboratory conditions to evaluate the effect of cell topology, base material, and polyurea reinforcement on the mechanical response of the structures. The experimental setups for quasi-static compression (Figure 6a), three-point bending (Figure 6b), and Charpy pendulum impact tests (Figure 6c) are shown schematically.

#### 2.4.1. Quasi-Static Compression

Quasi-static compression tests were performed using a Zwick/Roell universal testing machine with a maximum load capacity of 100 kN. Although no ASTM standard specifically covers the compression behavior of cellular structures, the test protocol was based on the principles of ASTM D695, which is commonly used for compression testing of polymeric materials. All specimens were compressed under displacement control at a constant rate of 5 mm/min until 50% of the nominal compression deformation was reached, allowing the elastic region, elastic limit, plateau stage, densification behavior, and recovery after unloading to be identified. The machine continuously recorded load and displacement data throughout the test.

Furthermore, auxetic and honeycomb cellular architectures have been widely described as highly effective systems for improving energy dissipation under mechanical loading due to their ability to undergo large plastic deformations without premature densification [48]. When combined with a surrounding matrix or secondary phase, these structures exhibit an extended deformation regime that promotes progressive collapse mechanisms, resulting in greater energy absorption efficiency. During compression loading, energy dissipation is primarily governed by the plateau region of the stress–strain response, where strain increases steadily while the stress level remains virtually constant [26,49]. This regime corresponds to controlled structural collapse and is particularly advantageous for impact-mitigation and protection applications, as it allows substantial energy dissipation without generating excessive reaction forces. To quantitatively evaluate energy absorption performance, considering differences in mass and material distribution, specific energy absorption (*SEA*) was used. This is defined as the energy absorbed normalized by the density of the specimen and is expressed in the Equation (1)

(1) $$SEA=\frac{1}{\rho }\times \int_{\varepsilon_{y}}^{\varepsilon_{d}}\sigma \left(\varepsilon \right)d\varepsilon$$

where ρ corresponds to the density of the structure, $\varepsilon_{y}$ represents the deformation corresponding to the onset of yield, and $\varepsilon_{d}$ indicates the deformation associated with the onset of densification. This formulation allows a direct comparison of the energy absorption efficiency of cellular structures with different topologies, materials, and reinforcement strategies, regardless of their absolute mass.

#### 2.4.2. Three-Point Bending

Three-point bending tests were performed using the same 100 kN Zwick/Roell testing machine, following the general methodology described in ASTM D790. The tests were performed under displacement control at a constant loading rate of 5 mm/min. The specimens were supported with a span length of 100 mm as shown in Figure 6b, ensuring that the central unit cell, highlighted in Figure 2, was directly subjected to the applied load. This configuration was selected to promote a bending response representative of the cellular architecture while minimizing contour effects. Force and displacement data were recorded and used to determine the flexural stiffness and related mechanical parameters.

#### 2.4.3. Charpy Impact Testing

Impact resistance was evaluated using Charpy pendulum impact tests performed with API Legacy High Performance equipment, with a maximum energy capacity of 4 J. The test procedure followed ASTM E23, serving as a reference framework for impact testing methodology and data interpretation. It must be explicitly noted that for highly damage-tolerant configurations, this 4 J equipment limit restricts the interpretation of the results; if a specimen does not fracture, the apparatus records a lower-bound estimate rather than the true absolute impact strength. The specimens were placed on supports separated by 65 mm as shown in Figure 6c, ensuring that the central unit cell received the impact load. The absorbed impact energy ($E_{abs}$) was obtained from the difference between the initial and residual energy of the pendulum after fracture or deformation. This configuration enabled consistent comparison of impact energy-absorption performance across different cell configurations and material systems.

During the Charpy impact tests, the $E_{abs}$ was provided directly by the impact pendulum, corresponding to the difference between the initial energy and the residual energy of the pendulum after fracture or deformation of the specimen. This measured energy represents the total energy dissipated by the specimen during the impact. Based on the absorbed energy values, the impact resistance was calculated by normalizing the absorbed energy with respect to the effective cross-sectional area of the specimen in the impact section, according to the Equation (2)

(2) $$a_{c}=\frac{E_{abs}}{A}$$

where $a_{c}$ is the Charpy impact strength in kJ/m^2^, $E_{abs}$ is the absorbed energy in Joules (J), and *A* is the cross-sectional area of the specimen in mm^2^, allowing for a direct comparison of impact energy absorption efficiency between different cell configurations and material systems. While absorbed energy provides an absolute measure of the energy dissipated during impact, Charpy impact resistance allows for a standardized comparison of impact performance by accounting for differences in specimen geometry. This parameter is particularly relevant when comparing cellular structures with different dimensions, densities, or topologies, as it reflects the intrinsic impact resistance per unit area, rather than the total energy absorbed.

The following section presents the experimental results obtained for cellular structures made of *ABS-FRO* and *PA-GF*, both in their unreinforced and polyurea-reinforced states, highlighting the influence of material selection and elastomeric reinforcement on the overall mechanical response, including Young’s modulus, yield strength, specific energy absorption (*SEA*), flexural stiffness, and impact strength.

## 3. Results and Discussion

In accordance with the experimental procedures described in the previous section, this section presents and analyzes the mechanical performance of cellular structures additively manufactured with *ABS-FRO* and *PA-GF* filaments, evaluated both without reinforcement and with polyurea elastomer. For each Auxetic-Auxetic (A−A), Honeycomb-Honeycomb (H−H), and hybrid configurations (A−H and H−A), independent sets of specimens were tested under quasi-static compression, three-point bending, and Charpy impact loading, allowing for a comprehensive evaluation of their structural response under different loading regimes.

The results presented in the following subsections focus on key mechanical indicators, including Young’s modulus, yield strength, specific energy absorption (*SEA*), flexural stiffness, impact resistance, and, in some cases, post-test recovery. Particular attention is paid to the role of cellular topology, base material selection, and polyurea reinforcement in regulating deformation mechanisms, energy dissipation efficiency, and damage tolerance. This integrated analysis provides key insights into the performance of hybrid architectures for advanced energy absorption and impact mitigation applications.

### 3.1. Quasi-Static Compression Response

**Figure 7:**
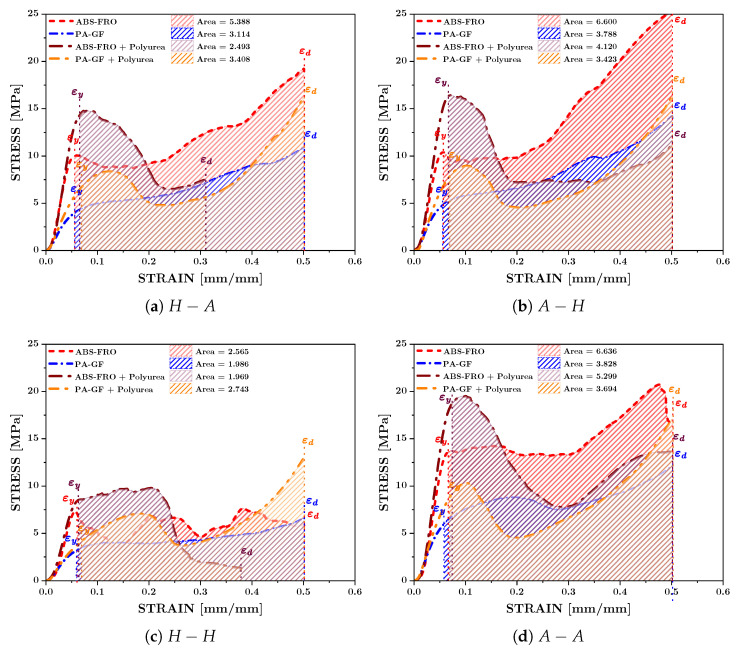
Stress vs. Strain response under quasi-static compression for the four cellular topologies evaluated: (**a**) H−A, (**b**) A−H, (**c**) H−H, and (**d**) A−A. Each plot compares the mechanical behavior of specimens printed with *ABS-FRO* and *PA-GF*, as well as their corresponding polyurea-reinforced versions, highlighting the effects of material selection and elastomeric coating on stiffness, collapse behavior, and energy absorption.

The stress–strain curves for the hybrid H−A and A−H configurations (Figure 7a,b) show intermediate behavior. In contrast, the pure H−H and A−A responses (Figure 7c,d) highlight the topological extremes of the studied design space. The results reveal a multi-stage deformation behavior characteristic of cellular architectures, consisting of an initial linear elastic region, followed by yielding and a collapse plateau, and finally a densification stage at higher deformations. The shaded areas under the stress–strain curves shown in Figure 7 represent the energy density of deformation absorbed during compression (∫σdε), expressed in units equivalent to [MPa] (1 MPa = 1 MJ/m^3^). These values were subsequently normalized by sample density to calculate the Specific Energy Absorption (SEA) in [J/g]. A clear influence of both cell topology and material selection on the response to compression is observed. In general, samples made with *ABS-FRO* exhibit higher initial stiffness and yield stress compared to their *PA-GF* counterparts, reflecting the intrinsic mechanical properties of the base material. Furthermore, incorporating polyurea significantly alters the post-yield response, promoting a more stable, extended plateau and delaying the onset of densification. This behavior indicates greater energy-dissipation capacity and improved damage tolerance, especially in hybrid and auxetic configurations. It should be noted that the differences between topologies are accentuated in the plastic regime, where deformation mechanisms are governed by strut buckling and progressive collapse. In standard FDM structures, this collapse is heavily exacerbated by weak interlayer adhesion, which acts as a site for crack initiation. This significant improvement in yielding and post-yield stability is attributed to the ’hydraulic confinement’ effect provided by the polyurea matrix. Unlike traditional porous foams that collapse under low pressure, the nearly incompressible nature of the polyurea elastomer allows it to act as a localized hydraulic jacket around the cellular struts. During compression, as the FDM struts begin to yield or buckle, the surrounding polyurea generates a multi-axial resistive pressure that redistributes stress concentrations away from the weak interlaminar FDM interfaces. However, the hybrid configurations (H−A and A−H) exhibit intermediate responses between purely auxetic and honeycomb structures, suggesting a synergistic interaction between the two cellular topologies. These results highlight the combined role of geometry and elastomeric reinforcement in adapting the compression performance and energy absorption characteristics of the proposed cellular systems.

A direct comparison between purely honeycomb (H−H) and purely auxetic (A−A) configurations reveals pronounced differences in their compression deformation mechanisms and energy-absorption capacities. The H−H structures consistently exhibit the lowest initial stiffness and elastic limit among the evaluated topologies, regardless of the base material or the presence of polyurea reinforcement. This reduced capacity is associated with premature cell wall bending and localized buckling, which promotes premature structural instability [50]. Even when reinforced with polyurea, several H−H samples collapse prematurely before reaching 50% compression deformation, indicating limited plastic deformation capacity and reduced effectiveness of the elastomeric phase in stabilizing the fracture process. In contrast, the A−A configurations demonstrate a markedly superior mechanical response under quasi-static compression. These structures exhibit the highest initial stiffness and yield stress, reflecting the load transfer efficiency inherent in auxetic architectures. The negative Poisson’s ratio promotes lateral densification during compression, leading to progressive cell interaction and greater structural restraint [51]. As a result, the A−A samples exhibit a more uniform and extended plateau region, particularly in the polyurea-reinforced condition, allowing sustained plastic deformation without sudden stress drops. This behavior results in significantly higher energy absorption, as evidenced by the larger area under the stress–strain curve.

In this way, the different mechanical responses of the H−H and A−A structures generate a contrast that highlights the role of cellular topology in manipulating stiffness and post-yield stability [52]. While honeycomb architectures favor early deformation through mechanisms dominated by bending, auxetic structures activate collapse modes dominated by strut stretching and rotation, which couple more effectively with elastomeric reinforcement [53,54]. Consequently, the A−A configuration stands out as the most efficient topology for quasi-static compression in energy-absorption applications, offering superior mechanical stability, delayed densification, and greater plastic deformation capacity when combined with polyurea.

Table 2 presents the deformation sequence of polyurea-reinforced cellular structures made of *PA-GF* under quasi-static compression, capturing the representative stages of the stress–strain response, including yielding, plateau, maximum deformation, and recovery after unloading. This specific subset of samples is presented because elastic recovery was predominantly observed in polyurea-reinforced *PA-GF* structures. In contrast, other material combinations and topologies showed negligible or very limited recovery after unloading. This pronounced difference in recoverability is attributed to the intrinsic post-yield deformation mechanisms of the base polymers. ABS undergoes irreversible plastic yielding and craze-formation under high compressive strains, which permanently deforms the cellular skeleton [55]. Conversely, the semi-crystalline nature of the polyamide matrix, reinforced by glass fibers, allows the PA-GF struts to retain a higher degree of structural integrity post-yield [56,57]. When infiltrated with the highly resilient polyurea elastomer, the PA-GF lattice successfully leverages the elastomer’s elastic restoring force to spring back. In contrast, the plastically yielded and micro-cracked ABS framework permanently restricts macroscopic recovery. Consequently, the deformation sequences shown in this table focus exclusively on configurations where this behavior was clearly identifiable and mechanically relevant. In addition to illustrating the collapse mechanisms, Table 2 reveals a distinctive recovery capacity induced by the polyurea reinforcement in combination with the *PA-GF* network. After unloading, all evaluated configurations exhibit partial height recovery, indicating that part of the deformation energy stored during compression is released elastically. This response highlights the active role of the elastomeric phase in stabilizing the cellular architecture and promoting reversible deformation mechanisms at high strains.

A quantitative comparison of the deformed heights before and after unloading reveals clear differences between the evaluated topologies. For hybrid configurations, recovery rates of 78.7% and 77.4% were obtained for the H−A and A−H structures, respectively, indicating partial but consistent elastic recovery thanks to the polyurea matrix. The predominantly honeycomb configuration H−H exhibits a slightly higher recovery level of 81.0%, suggesting that although this topology experiences pronounced local buckling during compression, the presence of polyurea contributes to a moderate restoration of the overall geometry. In contrast, the auxetic configuration A−A demonstrates significantly higher recovery, reaching 96.6% of its original height after unloading, confirming its exceptional ability to withstand large deformations while preserving structural integrity. The significantly improved recovery exhibited by the A−A topology can be attributed to the interaction between the negative effect of the Poisson’s ratio and the elastic resilience of the polyurea reinforcement. During compression, the auxetic architecture promotes lateral contraction and progressive densification, allowing deformation energy to be distributed more evenly and stored efficiently within the elastomeric phase. Upon unloading, this stored energy is released, propelling the structure back to its initial configuration. Hybrid architectures H−A and A−H exhibit intermediate levels of recovery, reflecting the coexistence of auxetic-driven deformation and localized honeycomb collapse mechanisms. Overall, these results demonstrate that polyurea reinforcement not only improves energy absorption during the plateau stage but also introduces a recoverable deformation capacity, particularly when combined with auxetic architectures, making these systems highly attractive for reusable energy absorption and impact mitigation applications.

### 3.2. Flexural Stiffness Response

**Figure 8:**
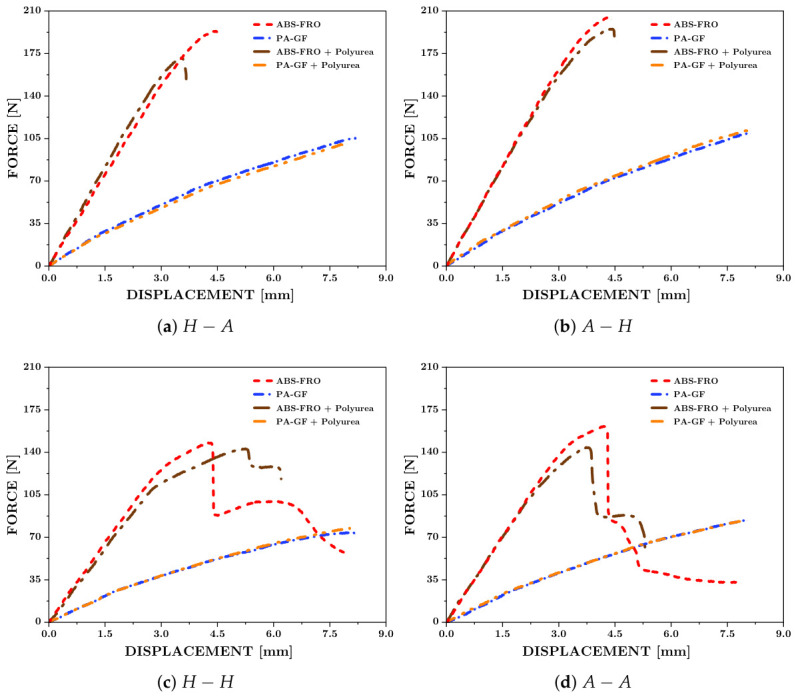
Force vs. Displacement response under three-point bending for the four cellular topologies evaluated: (**a**) H−A, (**b**) A−H, (**c**) H−H, and (**d**) A−A. Each plot compares the mechanical behavior of specimens printed with *ABS-FRO* and *PA-GF*, as well as their corresponding polyurea-reinforced versions.

The flexural behavior of the cellular structures was evaluated through three-point bending tests to assess their load-bearing capacity, stiffness, and deformation. The force–displacement curves for bending in the hybrid (Figure 8a,b) and pure topologies (Figure 8c,d) demonstrate consistent mechanical trends across loading modes.

Looking specifically at the raw data in Figure 8, a clear and consistent difference in flexural response is observed between the two base materials. In all cases, structures made from *ABS-FRO* exhibit a significantly steeper initial slope in the force vs. displacement curves, indicating significantly higher flexural stiffness compared to PA-GF samples. Quantitatively, the flexural stiffness of *ABS-FRO* structures is approximately three times greater than that of *PA-GF* structures, demonstrating the dominant influence of the intrinsic elastic properties of the base material over the cellular geometry in the dominant flexural regime. Furthermore, the incorporation of polyurea reinforcement does not result in a noticeable change in the initial flexural stiffness across any of the configurations evaluated. The force vs. displacement curves of the reinforced and unreinforced samples remain practically parallel in the elastic region, suggesting that the elastomeric phase contributes minimally to the load deformation under bending. This behavior can be attributed to the relatively simple beam design adopted in this study, which consists of a single unit cell thickness along its section. Under these conditions, the bending stresses are mainly supported by the outer cell walls. In contrast, the polyurea, located inside cell cavities, experiences limited deformation and therefore plays a marginal role in the elastic bending response.

Furthermore, the relationship between clear span and thickness and the localized deformation of the central cell where the load is applied can further reduce the mechanical involvement of the elastomeric reinforcement during bending. As a result, the structural response in the elastic regime remains largely determined by the stiffness of the printed polymer network rather than by the presence of polyurea. Although the elastomeric reinforcement is expected to improve post-yield stability and damage tolerance by suppressing crack initiation and propagation, ultimate fracture resistance could not be conclusively evaluated in the present study, as the flexural tests were performed up to 50% of the imposed displacement, in accordance with the adopted test protocol, without reaching catastrophic failure. Thus, these results indicate that, while material selection plays a key role in determining flexural stiffness, the influence of polyurea reinforcement under flexure depends largely on structural design and loading mode. It must be explicitly noted that these flexural tests were conducted on single-cell-thick profiles to isolate the localized bending response of the outermost struts. Consequently, the internal polyurea matrix contributed minimally to the initial flexural stiffness. Future evaluations of multi-layer bulk composites are required to fully characterize the macro-scale flexural toughness and the three-dimensional elastomer–skeleton interaction.

**Figure 9:**
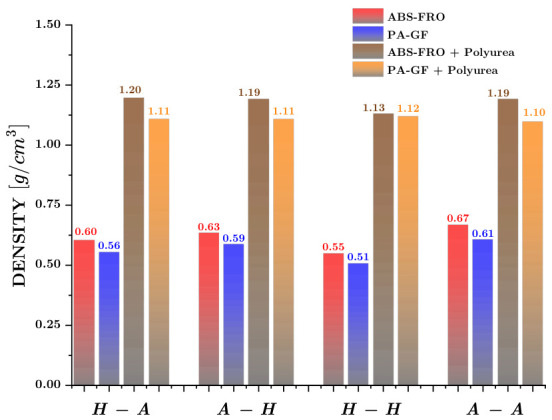
Density comparison of all cellular configurations fabricated using *ABS-FRO* and *PA-GF*, including their corresponding Polyurea-reinforced structures. The figure highlights the effect of cellular topology and elastomeric reinforcement on the overall density of the specimens.

Figure 9 presents a comparative analysis of the densities of all evaluated cellular configurations manufactured with *ABS-FRO* and *PA-GF*, including their corresponding polyurea-reinforced counterparts. As expected, incorporating the polyurea elastomeric matrix increases the total mass of the samples, resulting in higher densities than those of unreinforced structures for all cellular topologies.

**Figure 10:**
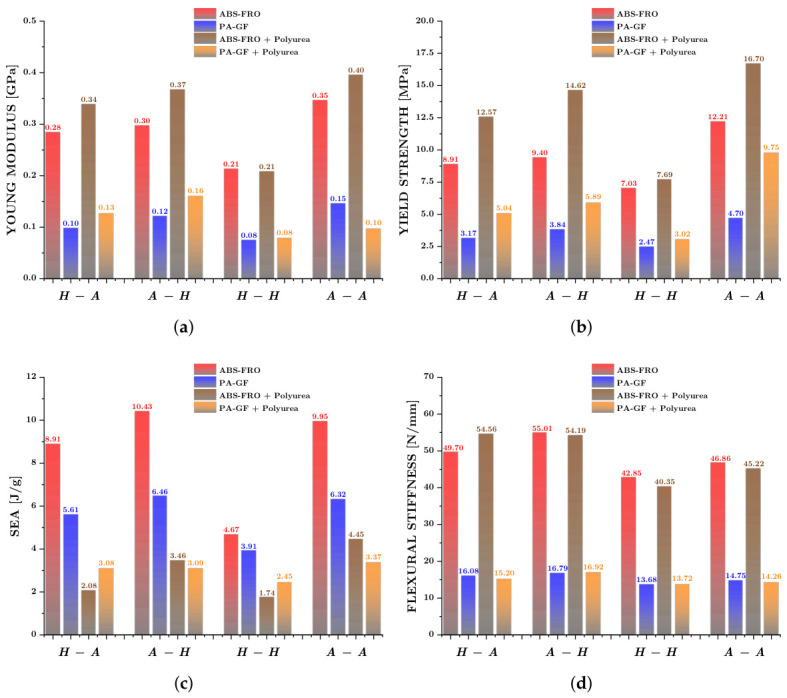
Comparative mechanical properties of the cellular structures for all evaluated configurations. The subfigures present: (**a**) Young’s modulus, (**b**) yield strength, (**c**) specific energy absorption (*SEA*), and (**d**) flexural stiffness for specimens fabricated using *ABS-FRO* and *PA-GF*, as well as their corresponding polyurea-reinforced structures.

This density variation is a critical factor that must be explicitly considered when evaluating and comparing the mechanical performance of different configurations. Since polyurea reinforcement can improve energy dissipation, deformation stability, or damage tolerance, the associated increase in mass can distort direct comparisons of metrics based on force, stress, or energy if density effects are ignored. Therefore, quantifying the density of each sample allows for a normalized evaluation of mechanical properties, enabling a more objective comparison of structural efficiency between materials and geometries. In particular, density normalization is essential for calculating specific mechanical metrics, such as specific energy absorption (*SEA*), which relates absorbed energy to the material’s mass or density. By incorporating density into the analysis, the influence of cell topology, base material selection, and elastomeric reinforcement on energy absorption performance can be evaluated, independent of weight penalties. Mechanical properties, including Young’s modulus, yield stress, density-normalized energy absorption (*SEA*), and stiffness, are analyzed in Figure 10, providing a comprehensive comparison of the structural efficiency of the proposed cellular systems.

In terms of elastic response, Figure 10a shows that cellular structures made from *ABS-FRO* have, on average, Young’s modulus values approximately 2.5 times higher than those of their *PA-GF* counterparts, regardless of cellular topology. This behavior is consistent with the intrinsic stiffness of *ABS*-based filament and its greater resistance to elastic deformation under compressive loading. From a geometric perspective, the predominantly auxetic A−A configuration consistently exhibits the highest elastic modulus, while the honeycomb H−H configuration exhibits the lowest stiffness, reflecting the improved load redistribution and lateral restraint provided by the auxetic architecture. The introduction of polyurea produces only a slight increase in Young’s modulus, approximately 12.8% for ABS-FRO and 4.5% for PA-GF, suggesting that elastomeric reinforcement plays a secondary role during the elastic regime, where deformation is governed primarily by the stiffness of the polymer struts rather than the flexible matrix.

A similar trend is observed for the elastic limit, as shown in Figure 10b, where the A−A structures have the highest yield stress and the H−H configuration has the lowest. Unlike the elastic response, polyurea reinforcement has a pronounced effect on the onset of plastic deformation, increasing the yield strength by approximately 27% for *ABS-FRO* structures and up to 40% for *PA-GF* structures. This significant improvement can be attributed to the confinement effect provided by the elastomeric matrix, which delays local buckling of the cell walls, redistributes stress concentrations, and promotes a more stable transition from elastic to plastic deformation [58]. The greater relative improvement observed for *PA-GF* structures suggests that polyurea effectively compensates for the lower intrinsic strength of the base material.

Furthermore, the energy absorption behavior under quasi-static compression, quantified through the area under the stress–strain curves, follows the same structural trend, Figure 10c. The configurations dominated by auxetics A−A exhibit the highest energy absorption capacity, while the honeycomb structures H−H show a reduced capacity to dissipate energy due to early localized collapse and limited plastic deformation. The hybrid configurations A−H and H−A exhibit an intermediate response, combining auxetic-induced lateral contraction with honeycomb-driven deformation mechanisms, thereby achieving a balanced compromise between stiffness and energy dissipation. Although polyurea reinforcement does not necessarily increase the total energy absorbed, it significantly stabilizes the plateau region, leading to a more homogeneous and controlled energy absorption process. This behavior is particularly relevant for impact mitigation applications, where avoiding force spikes is as critical as maximizing the energy absorbed. However, when incorporating density effects by calculating specific energy absorption (*SEA)*, a different perspective emerges. After normalization by density, *ABS-FRO* structures exhibit *SEA* values approximately 34% higher than those of *PA-GF* structures, indicating greater energy absorption efficiency per unit mass. Interestingly, among all configurations, the hybrid A−H topology achieves the highest *SEA*, suggesting that this architecture provides an optimal balance between structural mass, deformation stability, and energy dissipation mechanisms. Furthermore, density normalization reveals that unreinforced structures outperform their polyurea-reinforced counterparts in terms of *SEA*, with increases of approximately 65% for *ABS-FRO* and 46% for *PA-GF*. While increasing the strut thickness of the unreinforced *PA-GF* lattice could theoretically match the mass of the polyurea-infiltrated structure and potentially increase absolute energy absorption, it would not alter the fundamental failure mechanism. Thick-strut FDM polyamides still fail via catastrophic brittle fracture under impact due to layer-induced stress concentrations. The polyurea infiltration is utilized not to optimize mass-specific energy, but to provide hyperelastic hydraulic confinement. This elastomer acts as an incompressible hydraulic jacket around the brittle struts, restricting outward buckling, distributing interfacial stresses, and suppressing brittle fracture, thereby enabling the observed elastic recovery.

Finally, Figure 10d shows that flexural stiffness follows trends consistent with the Young’s modulus results, confirming the dominant influence of both the base material stiffness and the cellular topology on the flexural response. In general, structures made from *ABS-FRO* exhibit flexural stiffness values approximately 68% higher than those of their *PA-GF* counterparts, highlighting the decisive role of the material’s elastic properties under three-point bending. From a geometric point of view, the hybrid A−H configurations provide the highest flexural stiffness, while the H−H structures with a predominance of honeycomb remain the least rigid. It should be noted that the difference in stiffness between the A−H and A−A configurations is relatively small, with the hybrid topology being only 5.6% stiffer, indicating that auxetic mechanisms largely control the response to bending. In this flexural context, the contribution of polyurea reinforcement to flexural stiffness is practically negligible. In *ABS-FRO* structures, stiffness remains virtually unchanged after reinforcement, while in *PA-GF* samples only a marginal increase of approximately 2% is observed. This limited influence can be attributed to the elastomer’s flexible nature and the simplified beam design, which consists of a single unit-cell thickness, where flexural stiffness is determined primarily by the axial stiffness of the polymer struts rather than by the surrounding matrix. However, while polyurea does not significantly improve quasi-static flexural elastic stiffness, its beneficial role is expected to be accentuated under dynamic loading conditions, particularly in terms of impact resistance and damage tolerance, which are addressed in the next section.

It must be noted that the three-point bending tests were conducted up to a predefined displacement limit, resulting in partial deformation of the cellular core without reaching complete catastrophic fracture for most configurations. Consequently, while the polyurea infiltration undeniably enhances the structural integrity of the lattice, these benefits are strictly characterized herein as improvements in flexural stability, delayed localized yielding, and pre-fracture energy absorption capacity. Conclusions regarding the ultimate flexural fracture resistance or complete structural severing would require extended displacement testing, which falls outside the scope of the current quasi-static bending evaluation.

### 3.3. Charpy Pendulum Impact Resistance

The impact resistance of cellular structures is a critical parameter for applications involving dynamic loads, where the ability to dissipate energy quickly is essential, as catastrophic failures limit the structure. In this subsection, the impact response of auxetic, honeycomb, and hybrid cellular configurations is analyzed using the Charpy pendulum to evaluate their energy absorption capacity and damage tolerance under high-deformation-rate conditions. The experimental results allow a direct comparison between structures made from *ABS-FRO* and *PA-GF*, as well as their polyurea-reinforced counterparts, highlighting the role of cellular topology, base material, and elastomeric reinforcement in impact performance. Special emphasis is placed on the effectiveness of polyurea in improving impact energy dissipation and resistance to crack initiation and propagation, complementing the quasi-static mechanical behavior analyzed in the previous sections and providing a comprehensive assessment of the suitability of the structures for advanced energy absorption applications.

Table 3 summarizes the Charpy pendulum impact results obtained for all evaluated cellular topologies manufactured with *ABS-FRO* and *PA-GF*. The table presents the sample dimensions, absorbed impact energy, calculated impact resistance, and representative post-impact fracture morphologies, enabling a comprehensive comparison of dynamic energy-absorption performance as a function of cellular architecture and base material. A clear material-dependent trend is observed in all configurations. In general, *PA-GF* structures exhibit significantly higher impact energy absorption capacity than their *ABS-FRO* counterparts, with an average increase of approximately 70% in absorbed energy. This behavior is consistent with the greater intrinsic toughness and damage tolerance of glass fiber-reinforced polyamides, which promote crack deflection, progressive fracture, and better plastic dissipation under high strain rate loads [59].

Among the geometric configurations evaluated, the H−A configuration exhibits the highest impact resistance, especially when manufactured with *PA-GF*. It should be noted that the H−A sample of *PA-GF* did not fracture under the maximum available pendulum energy of 4 J, indicating an absorbed energy greater than the capacity of the equipment and a corresponding impact resistance greater than 40 kJ/m^2^. This excellent performance suggests a highly efficient energy-dissipation mechanism driven by the hybrid topology, in which the initial honeycomb-dominated region promotes controlled cell collapse and stress redistribution. In contrast, the auxetic region contributes to lateral restraint and delayed crack propagation.

The H−H topology also exhibits relatively high impact absorption capacity, reaching 0.82 J for *ABS-FRO* and approximately 2.55 J for *PA-GF*. The comparatively lower stiffness and elastic limit of this configuration, as described in Section 3.1 and illustrated in Figure 10, facilitate greater deformation before fracture, thus improving fracture toughness under impact loading. This response contrasts sharply with that of the predominantly auxetic A−A structures, which consistently show the lowest absorbed energy for both materials. The greater stiffness and elastic limit of the A−A topology, while beneficial under quasi-static compression, promote a more localized stress concentration and a comparatively brittle failure mode under impact conditions, limiting their energy dissipation capacity.

The hybrid configuration A−H exhibits intermediate behavior, balancing stiffness and impact resistance. Although its absorbed energy is lower than that of the H−A and H−H structures, it significantly exceeds that of the fully auxetic A−A topology, highlighting the advantages of combining auxetic and conventional honeycomb mechanisms to tailor impact performance. Overall, the impact results presented in Table 3 are fully consistent with the quasi-static mechanical trends analyzed previously. Structures with lower elastic stiffness and elastic limit tend to exhibit higher impact toughness and greater absorbed energy, whereas highly rigid configurations tend to favor premature fracture. These findings emphasize the crucial role of cellular topology in balancing stiffness, strength, and impact resistance. In particular, hybrid and honeycomb-dominant architectures, especially when combined with more tenacious base materials such as *PA-GF*, emerge as promising candidates for applications requiring efficient energy dissipation and high resistance to impact failure.

Table 4 presents the Charpy pendulum impact results for polyurea-reinforced cellular structures manufactured with *ABS-FRO* and *PA-GF* filaments. The table includes sample dimensions, absorbed impact energy, calculated impact resistance, and representative post-impact images, allowing direct comparison with the previously analyzed unreinforced structures and enabling evaluation of the contribution of the elastomeric coating under dynamic loading conditions. In line with the trends observed for unreinforced structures, polyurea-reinforced samples made from *PA-GF* exhibit significantly higher impact performance compared to those made from *ABS-FRO*. On average, structures reinforced with *PA-GF* exhibit at least 58% higher absorbed energy than their *ABS-FRO* counterparts. In particular, among the *PA-GF* specimens, only the fully auxetic A−A configuration experienced complete fracture, reaching an absorbed energy of approximately 2.77 J. In contrast, the remaining *PA-GF* configurations A−H, H−H, and H−A did not fracture under the maximum available pendulum energy, exceeding the 4 J capacity of the test device. The 4 J capacity of the Charpy apparatus was intentionally utilized as a threshold metric for catastrophic failure. The fact that polyurea-infiltrated *PA-GF* structures exceeded this kinetic energy limit without macroscopic rupture—whereas unreinforced structures catastrophically failed at roughly 2.0 J—serves as definitive experimental evidence of the failure mode transition from brittle fragmentation to stable elastomeric energy dissipation. However, as expected in hybrid prototyping, this absolute impact survival is accompanied by a density penalty. For example, while the absolute absorbed energy of the *PA-GF* A−A structure increased dramatically, its Specific Impact Energy (SIE) remained relatively low at 0.26 J/g compared to the 0.55 J/g of the unreinforced H−A structure. This explicit trade-off confirms that the triple-hybrid strategy prioritizes extreme damage tolerance and structural survivability over strict lightweight optimization.

Consequently, polyurea reinforcement leads to a 52% increase in absorbed energy for *ABS-FRO* structures, demonstrating that the elastomeric phase actively promotes critical energy dissipation mechanisms—such as localized stable deformation and crack deflection—while delaying macroscopic fracture under high-speed loads. However, as expected, this absolute increase is accompanied by a significant density penalty. For example, while the absolute absorbed energy of the *PA-GF A − A* structure increased by over 180%, its Specific Impact Energy (SIE) remained relatively low at 0.26 J/g compared to the 0.55 J/g of the unreinforced H−A structure (Table 3 and Table 4). This explicit trade-off confirms that the hybrid strategy prioritizes damage tolerance and structural survivability over strict lightweight optimization.

Overall, the dynamic impact results confirm that polyurea reinforcement significantly improves the mechanical performance of cellular structures, particularly when combined with higher-toughness base materials such as *PA-GF*. The mechanical coupling between the elastomeric matrix and the rigid cellular skeleton not only increases the total energy absorbed but also actively arrests catastrophic brittle fracture, ensuring a stable, damage-tolerant collapse. From a design perspective, these findings validate polyurea-reinforced auxetic–honeycomb architectures as a highly effective solution for advanced impact mitigation applications, where post-impact structural integrity and robustness under extreme dynamic conditions are primary performance requirements. However, several limitations of the current study must be acknowledged. First, because several PA-GF configurations successfully exceeded the 4 J maximum capacity of the available Charpy apparatus without catastrophic failure, their true absolute impact resistance rankings remain partially obscured, establishing only strict lower bounds. While the macroscopic transition from structural fragmentation to stable elastomeric survival has been definitively demonstrated, the ultimate energy dissipation limits require future quantification using higher-capacity equipment, such as a 50 J Charpy pendulum or instrumented drop-tower impact testing. Furthermore, while this work provides a robust experimental foundation, the absence of numerical or analytical modeling limits visualization of the complex internal stress redistribution at the skeleton–elastomer interface. Future research focusing on high-fidelity Finite Element Analysis (FEA) that incorporates the hyperelastic constitutive behavior of polyurea and the anisotropic properties of FDM struts will be essential to optimize these hybrid metamaterials. Finally, micro-scale fractographic and Scanning Electron Microscopy (SEM) analyses are required in future investigations to accurately validate the interfacial adhesion and crack-bridging mechanisms governing this macroscopic structural response.

## 4. Conclusions

This study demonstrated a hybrid composite methodology that overcomes the intrinsic layer-induced brittleness of FDM by programming failure mode transitions in polyurea-infiltrated auxetic–honeycomb architectures. By systematically evaluating these structures under quasi-static compression, flexure, and dynamic Charpy impact testing, this work establishes that topological sequencing and elastomeric confinement function as a robust Design for Additive Manufacturing (DfAM) strategy. The principal findings are summarized below:

- Cellular topology dictated the response to compression. The pure auxetic (A−A) configuration exhibited the highest stiffness, elastic limit, and energy absorption, supported by a stable plateau region.
- Polyurea infiltration significantly enhanced post-yield stability and recoverability. Elastic recovery reached 96.6% for the A−A *PA-GF* configuration, demonstrating an effective mechanical coupling between auxetic kinematics and elastomeric confinement.
- Flexural stiffness was governed by the base polymer. Polyurea had a negligible effect (< 2%) on the elastic flexural stiffness in single-cell beam designs, but it actively improved flexural stability and pre-fracture energy absorption beyond the elastic regime.
- *ABS-FRO* structures achieved *SEA* values 34% higher than *PA-GF*. While polyurea reinforcement reduced mass-specific energy efficiency due to the density penalty, it introduced a hyperelastic hydraulic confining phase essential for structural survivability.
- Dynamic impact testing confirmed the programmed failure mode transition. The H−A topology exhibited the highest impact tolerance, and polyurea infiltration increased impact resistance by 52% for *ABS-FRO* and by more than 30% for *PA-GF*, completely suppressing catastrophic brittle fracture.

These results demonstrate that integrating topological design with hyperelastic confinement effectively decouples structural stiffness from fracture toughness. By neutralizing the intrinsic layer-induced brittleness in FDM, polyurea-reinforced auxetic–honeycomb architectures offer a scalable, highly tunable solution for reusable, impact-survivable protective cellular composites.

## Figures and Tables

**Table 1:** Statistical summary of the mechanical properties obtained from five replicates of the A–H ABS-FRO reticular configuration, including mean value, standard deviation (SD), and coefficient of variation (COV).

Property	Average	SD	COV
Young’s modulus [MPa]	289.97	8.40	2.90%
Yield strength [MPa]	10.47	0.45	4.31%
SEA [J/g]	10.06	0.29	2.09%

**Table 2:** Deformation sequence of polyurea-reinforced cellular structures under quasi-static compression. The deformed height evolution is shown for configurations *H*–*A*, *A*–*H*, *H*–*H*, and *A*–*A* at key stages of the stress–strain response (yield, plateau, maximum strain, and post-unloading recovery). All specimens were fabricated using *PA-GF*.

	Yield Strain	Plateau Stage	Maximum Strain	Recovery
H−A	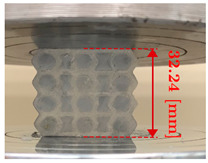	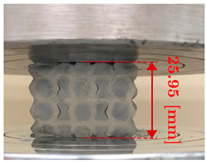	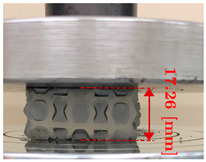	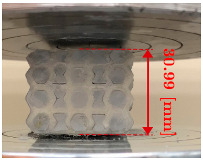
A−H	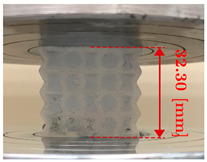	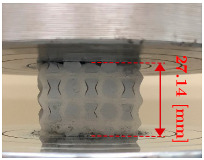	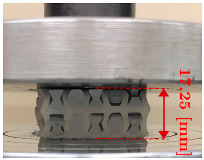	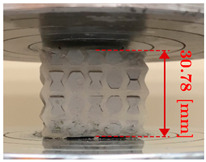
H−H	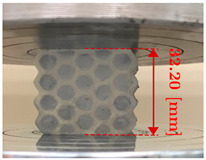	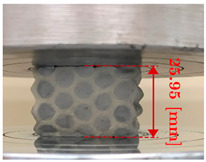	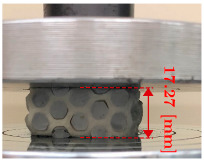	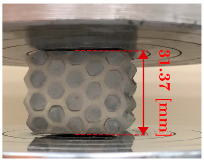
A−A	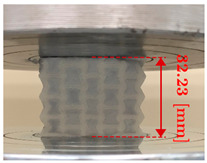	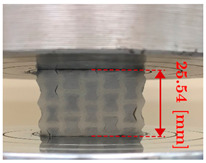	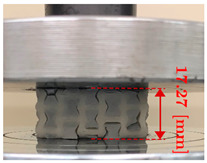	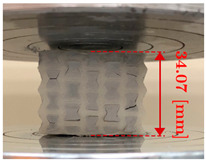

**Table 3:** Charpy impact results for each cellular topology and printing material, including specimen dimensions, absorbed energy, impact strength, and representative post-impact images.

	Material	Thickness (mm)	Width (mm)	Absorbed Energy (J)	Impact Strength (kJ/m^2^)	Sample
H−A	*ABS-FRO*	10.8	9.96	1.0389	9.658	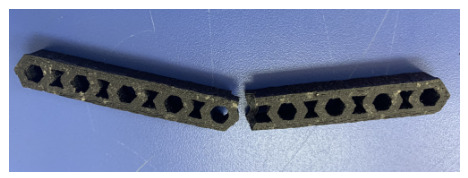
	*PA-GF*	10.25	9.70	> 4	>40	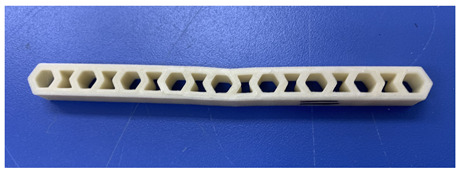
A−H	*ABS-FRO*	10.80	10.10	0.6089	5.582	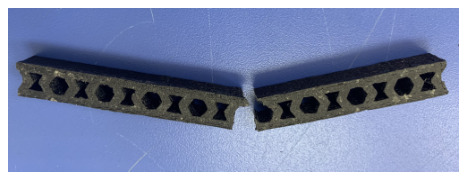
	*PA-GF*	10.37	9.66	2.2892	22.852	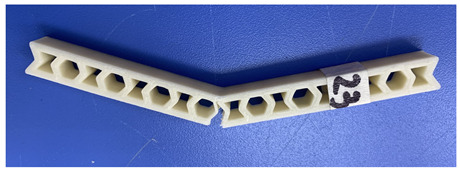
H−H	*ABS-FRO*	10.62	9.82	0.8181	7.845	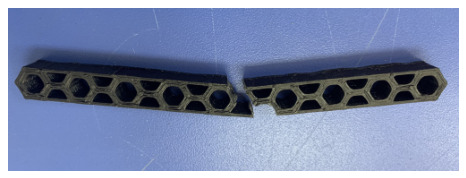
	*PA-GF*	10.24	9.54	2.5490	26.093	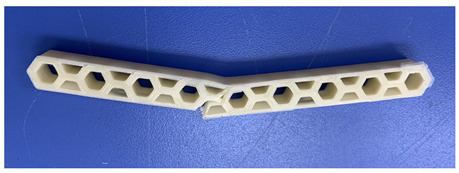
A−A	*ABS-FRO*	10.65	9.65	0.5748	5.593	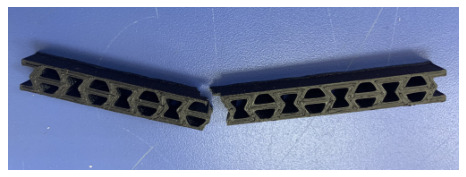
	*PA-GF*	10.17	9.70	1.3914	14.105	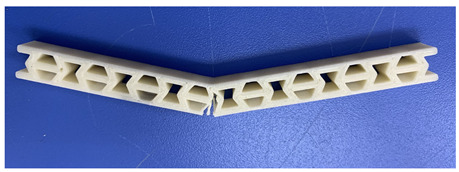

**Table 4:** Charpy impact results for Polyurea-reinforced cellular structures, including specimen dimensions, absorbed energy, impact strength, and representative post-impact images.

	Material	Thickness (mm)	Width (mm)	Absorbed Energy (J)	Impact Strength (kJ/m^2^)	Sample
H−A	*ABS-FRO*	10.57	9.59	0.8016	7.908	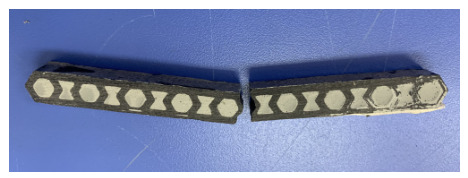
	*PA-GF*	10.57	10.3	>4	>37	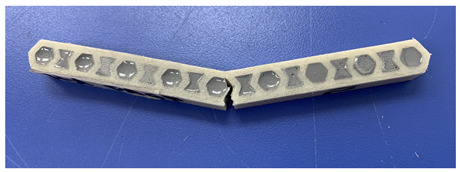
A−H	*ABS-FRO*	10.94	9.79	0.9959	9.299	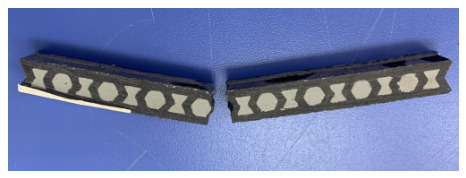
	*PA-GF*	10.56	10.30	>4	>45	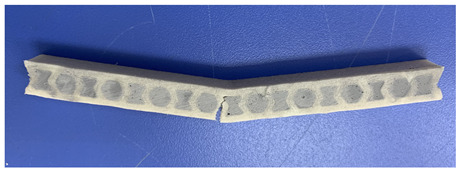
A−A	*ABS-FRO*	10.37	9.54	0.7375	7.455	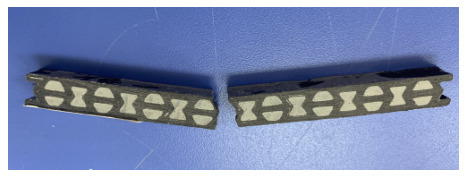
	*PA-GF*	10.31	10.20	2.7672	26.314	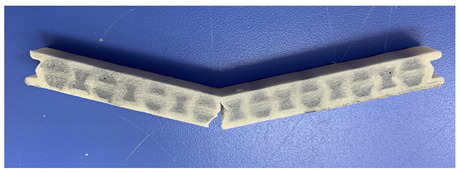
H−H	*ABS-FRO*	10.47	10.16	3.7374	35.134	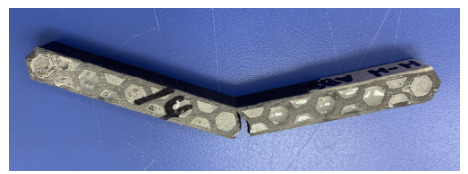
	*PA-GF*	10.39	10.32	>4	>37	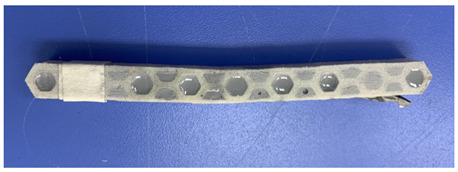

## Data Availability

The original contributions presented in this study are included in the article. Further inquiries can be directed to the corresponding author.
